# Effectiveness of a brief body-oriented intervention on muscle activities when playing the violin

**DOI:** 10.3389/fpsyg.2026.1944291

**Published:** 2026-10-14

**Authors:** Manfred Nusseck, Kilian Emslander, Claudia Spahn

**Affiliations:** Freiburg Institute for Musicians’ Medicine, University of Music Freiburg, Medical Center and Faculty of Medicine – University of Freiburg, Freiburg Center for Music Research and Teaching, Freiburg, Germany

**Keywords:** Feldenkrais method, intervention, motion analysis, muscle activity, playing position, playing-related problems

## Abstract

**Background:**

Violin performance requires highly complex sensorimotor coordination, resulting in specific playing techniques and individual ergonomic strategies. Despite this adaptability, violinists frequently experience musculoskeletal problems, particularly involving muscle fatigue and overuse. A better understanding of muscle activities during violin performance and approaches to reduce muscular load remains necessary.

**Objective:**

This study investigated the effects of a brief body-oriented intervention based on the Feldenkrais Method on muscle activity and movement patterns during violin performance.

**Methods:**

Fourteen violinists performed a musical excerpt while upper-limb kinematics, violin position, and electromyographic (EMG) activity of selected shoulder and arm muscles were recorded. Measurements were obtained at three time points: (1) baseline, (2) following a 30-min rest period, and (3) after a 45-min Feldenkrais-based intervention. Participants also reported the presence of playing-related musculoskeletal complaints.

**Results:**

The EMG activity values decreased across the three measurements in all muscle groups. However, the muscle activity of some muscle groups, including the forearm flexor muscles on both sides and the left biceps, decreased after the pause, while the muscle activity of other muscle groups, including both upper trapezius muscles and the right biceps, decreased after the intervention. The range of motion in the right arm remained unchanged across the measurements. The analysis of the violin positioning identified four distinct playing types based on the violin height (high: scroll at shoulder level; low: scroll at breast level) and angle relative to the frontal plane (approximately 20° or 45°). Certain muscle activation patterns differed between these playing types. Violinists playing in a lower violin position reported shoulder and neck complaints more frequently than those performing in higher playing positions.

**Conclusion:**

Although the short rest period reduced activity in several muscles, the Feldenkrais-based intervention provided additional reductions in muscles that did not respond to rest alone, particularly the upper trapezius muscles, a region commonly associated with playing-related pain in violinists. The findings suggest that approaches based on body-oriented interventions are necessary to selectively reduce muscular load during violin performance. Those interventions help optimize muscle function and potentially reduce the risk of long-term musculoskeletal problems.

## 1 Introduction

Playing an instrument requires a specific playing position and the execution of complex physical movements. Thus, playing extensively for long periods of time can lead to muscle strain which may result in Playing-Related Musculoskeletal Disorders (PRMD) or even an overuse syndrome (Shan and Visentin, 2003). Up to 93% of musicians were found to suffer from PRMD or musculoskeletal pain (Kok et al., 2016; Kochem et al., 2026). In professional musicians, high string players were particularly at higher risk of musculoskeletal pain than other instrumentalists, where 65 to 88% of the players described serious problems which interfere with their ability to perform (Steinmetz et al., 2015; Rensing et al., 2018). In a systematic review, several pathologies related to playing the violin have been recognized with PRMD as the most common problem (Zinn-Kirchner et al., 2023). Professional violinists often complain about shoulder pain (McCrary et al., 2016). Even in young musicians, 67% reported having musculoskeletal problems mainly in the hands, elbows and shoulders on both sides (Ranelli et al., 2011).

Possible causes of these problems could be attributed to the violin’s basic playing position, repetitive movements, a constrained playing position and varying dynamic and static muscle loads (Ackerman and Adams, 2004; Rensing et al., 2018). Specific playing performances even differ between standing and seated playing position with a smaller range of motion in the right arm than in the left arm while seated (Spahn et al., 2014). Also, in seated position when holding the violin, an increased rotation of the torso and specific shoulder adaptations on both sides become apparent in comparison to the seated position without instrument (Ohlendorf et al., 2018).

Efficient movement patterns can decrease unnecessary muscle use and may help prevent PRMD (Fjellman-Wiklund et al., 2004b). For that, the identification of individual muscle loads is necessary to identify factors influencing PRMD while playing the violin (Rensing et al., 2018). When playing an instrument, an ideal distribution of muscle activity within the functional chains involved is an important prerequisite for avoiding muscle fatigue and overuse complaints (Shan and Visentin, 2013; Spahn, 2015; Türk-Espitalier, 2016).

Several studies have examined the physical demands of playing the violin. Various biomechanical measurement methods were used for this purpose, with 3D motion capture for motion analysis and electromyography (EMG) for assessing muscle activity being the most commonly employed methods (Schemmann et al., 2018). Some studies even performed a conjunction of both EMG and motion analysis to recognize relationships between playing and internal loading intensity.

Using motion capture analysis, a foundational study of the violin’s right arm using a biomechanical model revealed that muscular effort increased with tempo and when playing on the lower strings (Visentin and Shan, 2003). Furthermore, while the load of the right shoulder was in relation to the string played, the loads on the elbow and the wrist were detached of the string. By analyzing the motions of eleven professional violinists (Shan and Visentin, 2003), the results showed that playing on different strings affected the movements of the right arm, but less of the left arm. They also found that the left shoulder remained rather static while playing the violin, whereas the right arm performed a wider variety of movement patterns. The left arm, however, performs note-related shifts. Looking at those shifts in particular, Visentin et al. (2015) found that the duration of these hand position changes were rather similar across different playing tempos. They concluded that the violinists seem to have developed an individual pacing of changing positions when playing the violin.

Another commonly used method for measuring muscle activity is the surface electromyography (EMG). With this non-invasive technique, data about physiological processes of muscles are captured by measuring electrical potentials generated when muscles are activated (Shan and Visentin, 2012). For instance, this technique was used to investigate the muscle activity of violinists when performing downward and upward strokes when playing a single note (Afsharipour et al., 2016). The right trapezius muscle showed higher muscle activities when playing upwards strokes than bowing downwards. In another study, 18 skilled violin players were recorded using surface EMG at various muscles of the upper body (Mann et al., 2021). Static, median, and peak levels of muscle activity during playing a standardized scale and a music piece were captured. The results revealed higher muscle activities in the left forearm than in the right forearm, while no differences were observed between playing a scale or a music piece. Though, female players had higher muscle activity than male players. In a subsequent analysis, Mann and colleagues (Mann et al., 2023) found that the left forearm muscles showed higher activities when the playing tempo was increased and when playing with vibrato. For the right forearm muscles, playing forte caused the most muscular activity. These findings were in line with several other studies concluding that during violin playing the left arm is considerably static while the fingers move over the fingerboard and the right arm is rather dynamic while moving the bow over the strings (Rensing et al., 2018; Schemmann et al., 2018).

A particularly important aspect when investigating the muscular demands in violinists is the influence of how the player positions the instrument. In a study of Hildebrandt and colleagues (Hildebrandt et al., 2021), they compared the muscle activation (EMG) in four different positions of the violin: when the violin was hold at 20° and 50° angle from the player’s front (central sagittal plane) and with 20° and 50° angle tilt to the front (horizontal plane). The authors found that the highest muscle activity was measured when the violin was positioned 20° from the front with 20° inclination and the lowest muscle activity when the violin was positioned 50° from the front. They used an experimental setting with a specific fixation device for the instrument to ensure the different playing positions. However, the authors did not explain why they used these positions in particular. This study was repeated with the viola and confirmed that the instrument positioning affected muscle activation (Margulies et al., 2026).

The main aim of these studies was to identify potential risk factors that causes musculoskeletal problems. In this line, there are several studies which compared muscle activity of the upper extremities using EMG between players with and without self-reported musculoskeletal problems (Schemmann et al., 2018). For instance, greater muscle activities were found in neck (upper trapezius muscles) and shoulder muscles (deltoid muscles) in violinists with complaints (Kok et al., 2018). Considering additional muscle groups, such as the forearm flexors and the biceps, Möller and colleagues (Möller et al., 2018) also found higher EMG values in the group with musculoskeletal problems. By looking at specific musculoskeletal symptoms, McCrary and colleagues (McCrary et al., 2016) found that the right upper trapezius muscle activity was lower in participants who reported right shoulder problems. Players with right wrist symptoms showed higher muscle activities in all investigated muscles. In addition, the muscle activity of the left upper trapezius and other neck muscles on both sides were greater when players reported general neck pain (Park et al., 2012).

These studies showed that there are a variety of aspects which affect muscle activities of violinists. In a literature review, the musculoskeletal demands for violinists were described as complex for both left and right side but in different ways (Rensing et al., 2018). With all these studies in mind, violin performances should be thought of more as an individual process than as a kinematic reconstruction of the notes printed on the page of music (Shan et al., 2017). Investigating relationships between position, movements and internal loading intensities facilitate groundworks for developing injury prevention strategies and interventions (Visentin and Shan, 2003; Rensing et al., 2018).

Several studies investigated the effects of specific body-oriented interventions on various playing-related aspects such as musculoskeletal problems and pain in musicians (Laseur et al., 2023). For instance, an 8-week musculoskeletal health education program showed considerable positive effects on the dynamic spine function of music students (Adamčák et al., 2025). When performing 2 weeks daily warm-up exercises, perceived pain intensity of music students were reduced, but only when controlling for specific factors such as the physical activity of the students (Austen et al., 2024).

For violinists, an 8-week body awareness training program were conducted by five professional orchestra violinists (Fjellman-Wiklund et al., 2004a). In comparison to nine violinists without such a training, no considerable effects were found in the trapezius muscle activation, but there was a positive effect on the general muscle tension. However, a systematic review focusing on effects of motor control-based interventions on pain and range of motion in bowed string instruments (Adamik et al., 2026) found only limited evidences of positive effects of those interventions. The authors complained of the small number of studies and concluded that more research in this area is needed.

Body-oriented methods can be useful to support the awareness of muscle activity and of movement coordination (Spahn, 2017). The Feldenkrais Method is a technique of bodywork involving Functional Integration, with a focus on integrating body sensations and awareness with movement (Berland et al., 2022). Functional Integration, as an individual treatment within the Feldenkrais Method, is known by this term in reference to its approach and is described in the literature (Rywerant, 2003). It is a neuroscience-based method in which the therapist uses tactile aids and passive joint movements to provide the brain of the client with new sensory information, which in turn stimulates the brain to develop new, more economical, and better-coordinated movement patterns. In clinical applications, the method is often chosen to aid with recovery from injury, manage chronic conditions, or enhance the musculoskeletal system (Hillier and Worley, 2015). For instance, in elderly people the method can improve gait, balance, and mobility. Patients treated with the Feldenkrais Method often report changes in their body perception and an increased sense of relaxation, which has been confirmed by an increase in body pressure on the mat when lying on their back (Brummer et al., 2018). Several other studies also showed clear therapeutic effects of this method and a meta-analysis confirmed the positive intervention effect on the time to get up in older persons (Berland et al., 2022).

For individual treatment in musicians’ medicine, Feldenkrais Method is used for improving coordination of playing movements (Noé, 2017). Musicians experience their playing movements through a different body awareness of muscle tension in individual parts of the body and through a lightness of movement which have positive effects on playing technique and sound (Spahn, 2017). However, research into the effects of the Feldenkrais Method on muscle activity and movement patterns in musicians is still limited.

### 1.1 Purpose of the study

This study investigated how a short individual intervention using the Feldenkrais Method affects muscle activity in the upper body regions of violin players. The goal of the intervention was to enable the experience of the connection between hands, arms, shoulders and shoulder girdle with the spine and legs through sensory awareness. Effects of the intervention were assessed using motion analysis and surface Electromyography (EMG) across multiple measurements. In addition, various playing positions in accordance to Hildebrandt et al. (Hildebrandt et al., 2021) were identified as part of an exploratory design and used to analyze for differing effects.

The research followed the questions of (1) how average muscle activity in the upper-body muscles change following a short body-oriented intervention, (2) if there were differences in muscle activity between players with musculoskeletal complaints and musicians without complaints, and (3) if different playing positions differ and influence the effect of the intervention on muscle activity.

## 2 Methods

### 2.1 Participants

Fourteen professional violin players (8 female, 6 male, mean age 31.7 years between 19 and 61 years) were included. There was no significant difference in the mean age between genders. Six musicians were students and eight were working in orchestras or at music schools. The musicians estimated that they perform on average about 59 times a year, with the orchestra musicians performing more often (about 100–130 times) than the students (about 20 times). All players were trained in the modern style of playing the violin. None of them were regular baroque players.

Half of the players (7 out of 14) stated that they had health issues affecting their playing. These were all physical problems. In contrast, about 70% of the players (10 out of 14) reported experiencing pain and/or stiffness in the upper body regions. Six players located the source in their shoulders and/or the neck, while four players had problems in their arms or wrists. Considering both sides, the left shoulder and the left arm were mentioned most frequently. No players were excluded due to severe musculoskeletal problems.

### 2.2 Measurements

#### 2.2.1 Motion analysis

For the motion capturing, wireless IMU (Inertial Measurement Unit) sensors (WaveTrac, Cometa) were used. Each sensor contains a three-axis angular velocity gyroscope. Data were recorded with the Wave Plus receiver (Cometa) and the EMG & Motion Tools Software.

Nine IMU sensors were attached to the backs of both hands, both forearms, both upper arms, the nape of the neck (C7 vertebra), the sternum, and the forehead. These placements made it possible to determine the relative position of the upper body’s extremities. The data provided by the software included angle values with 2 DOF (degree of freedom) for the wrist, 1 DOF for the elbow and 3 DOF for the shoulder on each side.

Using these angles, rotation matrices for shoulder, elbow and wrist were created. In addition, for each segment (upper arm, forearm, wrist), a translation along the respective axis was defined in a translation matrix with unitary lengths. The rotation and translation were combined in a transformation matrix. The individual transformation matrices are multiplied in anatomical order: first the shoulder, then the elbow, then the wrist. With this total matrix, a kinematic reconstruction of the upper body was calculated for every time step. This procedure provides the spatial positions of specific joints based on the respective angles with same segment lengths across the players.

In Figure 1, schematic drawings of a violin player viewed from the front and from the top are shown including the movements of the elbow and the wrist of the right arm. The kinematic movement reconstruction was used to calculate the motion range of the elbow and the wrist. As seen in Figure 1, the elbow movements were mainly up and down motions restricted by the upper arm and the wrist follows the line of the bow mainly in right angle to the violin strings. For both the elbow and the wrist positions, the standard deviation of the movements, i.e., the distance to the mean position, was calculated in 3D. This provides a value of the motion range in the elbow and the wrist. These values have no unit because unitary segment lengths were used. They refer to the general motion range where higher values indicate greater movements. In addition to the spatial movements, the mean angle of the right elbow was included in the analysis.

**Figure 1:**
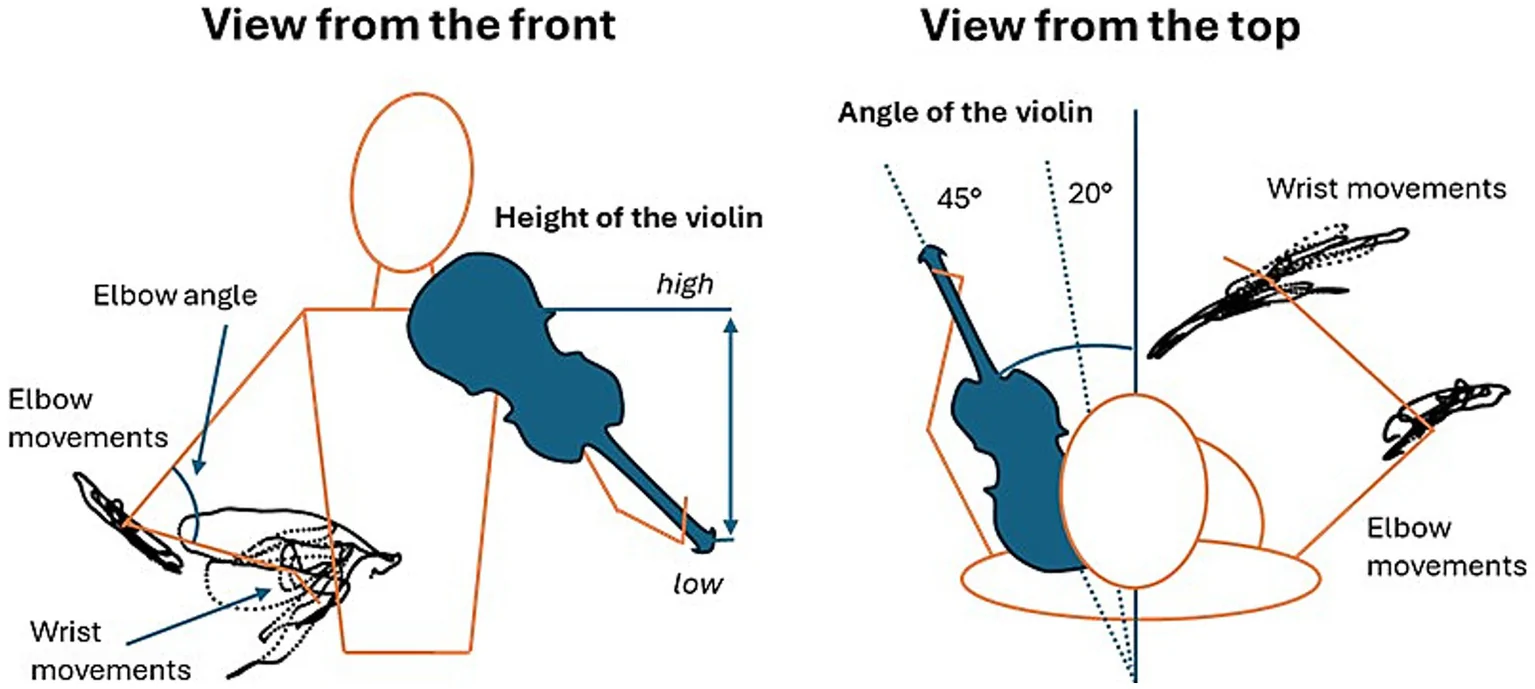
Schematic of a violin player with movement paths of the elbow and the wrist or the right arm.

Apart from the movements during position changes, there was only little movement in the left arm confirming the findings of other studies (Shan and Visentin, 2003). Therefore, the analysis of the movements in the left arm was not taken into account any further in the analysis. However, the position of the violin was determined from the left arm (Figure 1). Two values were extracted: (1) the height of the violin according to the shoulders (horizontal plane) and (2) the angle of the violin to the sagittal plane. The height of the violin was estimated relative to the shoulders. The violin position was defined as “high” when the instrument was held at a high position with the fingerboard nearly at shoulder level and “low” when the violin scroll was near breast level. The angle of the violin was also determined in relation to the front (central sagittal plane), i.e., it was zero degrees when the violin was pointing directly forward and increased the more the player held the violine to the left side.

#### 2.2.2 Electromyography

Wireless surface electromyography (EMG) sensors (Pico, Cometa) were used to measure muscle activity in arm and neck muscles during the musical performance. The sensors have 2000 Hz sampling frequency and were recorded with the same software (EMG & Motion Tools) as the IMU sensors. With that, both EMG and motion capture signals were synchronized.

Six EMG sensors were attached to the participants. They were placed at the arm muscles (forearm flexors and biceps) and the shoulder muscles (upper trapezius) on both sides. The chosen muscle groups are the primary muscles used in the chain of muscles when playing the violin (Visentin and Shan, 2003). The EMG sensors were placed parallel to the muscle fibers. The raw EMG signals were processed using a fourth-order Butterworth band-pass filter with cutoff frequencies of 20 and 450 Hz. The EMG data were than full-wave rectified by calculating the absolute values and smoothed using a low-pass filter to reduce high-frequency fluctuations. The mean overall value was calculated from this envelope curve representing the general muscle activation profile similar to the root mean square (RMS).

The EMG data are presented as mean values across all participants. These descriptive data were included solely to illustrate potential overall differences in activation between the individual muscle groups, as the EMG signals were not normalized and are therefore less suitable for direct between-subject comparisons. For the statistical analyses, the raw EMG values were used to assess within-subject differences across the measurement time points using repeated-measures analysis.

To enable interindividual comparisons of the EMG data, the Peak Dynamic Method (PDM) was applied for signal normalization. This approach normalizes raw EMG data relative to the highest muscular activity recorded during a task-specific dynamic movement, rather than to an isometric Maximum Voluntary Contraction (MVC). The resulting PDM value are expressed as the percentage of the mean muscle activity in relation to the highest performed activity. This method provides an indication of the relative muscle activation within the performed task. Lower values indicate that the mean muscle activity during the task was further below the individual’s peak recorded activation, whereas higher values reflect a greater proportion close to the maximal dynamic muscle activity.

In the results, the mean EMG values across all participants are reported along with the maximum activation value and the PDM value. In the Supplementary material, these values are shown for each player individually.

#### 2.2.3 Questionnaire

A printed questionnaire was given to the participants containing personal, musical and health-related questions. First, general questions about the person, such as age, gender and current occupation were asked. Musical questions considered the estimate of how often they perform in a year and how good they know and play the required piece.

For the health-related questions, the Epidemiological Questionnaire for Musicians (EPI) (Spahn et al., 2002) was used. In a question, players are asked whether they have any health issues that affect their playing (yes/no). If so, they can choose if they were primarily physical, psychological or both. In an additional question, the players were asked if they currently experience pain and/or stiffness in the upper body-regions (yes/no). The players were also asked to rate the pain level in different body regions on a 4-point scale (1: no pain; 4: very strong). The regions were the wrist, the arm and the shoulders separately of both sides as well as the neck and the upper back.

### 2.3 Procedure

The violinists were invited to participate in the study either personally or via mail. They were informed about the measuring procedure in detail in a letter of information. To be eligible to participate, potential participants needed to have several years’ experience playing the violin in an amateur or professional orchestra or ensemble. After agreeing to participate, they signed a written consent form upon arrival on the individually scheduled test day. The study was approved by the Ethical Commission of the University Clinic Freiburg.

The participants were notified in advance that they would have to play the opening of Bach’s Partita No. 2 in D minor. The sheet music laid on a music stand in front of the players. The duration of the excerpt was approximately one to one and a half minute. There was no metronome to set a specific tempo. About 80% of the participants (11 out of 14) reported in the questionnaire that they can play the piece good to very good.

On arrival, the EMG and motion sensors were attached to the participants. Subsequently, they had to play the piece three times (see Figure 2): (1) after the sensors were placed and the calibration was finished, (2) after a break during which they had to fill in the questionnaire and were allowed to read and (3) after the intervention. The second performance was intended to serve as an intra-subject control condition. The mean duration of the break was 31 min (SD = 5.5 min).

**Figure 2:**
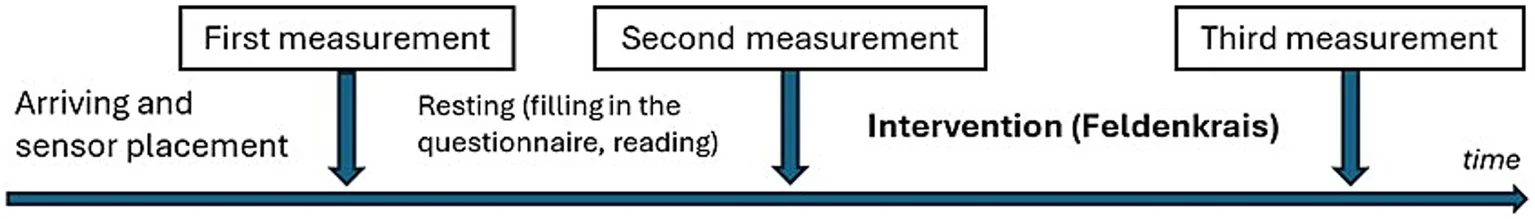
Schematic of the study design.

Before playing, the participants were measured once while standing without the instrument, with their arms at their sides, for about 10 s. This was a static baseline measurement to determine muscle tension at rest. Unfortunately, due to technical problems, the data of only 10 out of the 14 participants could be used for the analysis of the baseline condition.

### 2.4 The intervention

The body-oriented intervention lasted in total about three-quarters of an hour (46 min, SD = 5.2 min). At the beginning, the intervention leader welcomed the participant, explained the upcoming agenda of the intervention, asked them to what extent they already had experience with the Feldenkrais method and prepared them for the treatment. After the treatment, the participants were asked to provide some brief feedback. This procedure resulted in a real treatment time of approximately 30–35 min.

The intervention followed the principles of Functional Integration according to Feldenkrais. The aim of the intervention was to enable the subjects to experience the movement chain between the hands, arms, shoulders and shoulder girdle with the spine and legs in a sensorimotor way. The intervention was performed by a physiotherapist who was trained as Feldenkrais practitioner.

The player was positioned on the back on a treatment table. A firm foam roll with a diameter of 23 cm was placed under their knees and a smaller roll with a diameter of 12 cm was placed below the calves. This ensured that the weight of the thighs and legs did not exert pressure on the pelvis. A support was placed under the head to rest it comfortably in line with the spine and to avoid a tilt forward or backward. First, the therapist sat at the foot of the bed and applied a pressure impulse to both feet near the talus resulting in a slight “rolling motion” of the pelvis that continued along the spine toward the head. Afterwards, the therapist moved the arm of the player to the side and performed bending and straightening movements of the wrist and the fingers. Small rotation movements were applied to the fingers followed by the forearm and the upper arm. Then, the arm was moved through the shoulder joint in the various possible directions. This procedure was repeated with the other arm. The players had to passively follow the movement by perceiving the effects of these movements in the whole body. After that, the therapist stood at the head of the bed, grasped both arms at the wrists, and simultaneously moved the arms in various possible directions, not only symmetrically, but also asymmetrically. This provides a sense of the arm movements to understand how the arm movements are coordinated and controlled by the entire body. At the end, the players came back to the upright position perceiving the effects of the treatment. Verbal instructions were only used to explain the procedure and each individual step.

All participants were given the same treatment protocol described above using the Feldenkrais Method of Functional Integration. However, there were some individual variances which had to be adopted depending on the participant. Those considered the range of movement, the participant’s muscular tension and the intensity of the exercises performed. The practitioner regarded these variations as a means of achieving the intervention’s general aim.

In the short feedback, the players reported standing effortlessly, distributing their weight evenly across both legs and feet, having a lower center of gravity, relaxed shoulders, feeling taller, feeling in balance and feeling their hands and fingers very present. They also described having a deeper breathing, their arms feeling lighter and overall, both of their arms and their entire body to be much more relaxed.

### 2.5 Analyses

For the analyses, the first two measures of the piece were included. This section was about 10 s long. The playing on each string and the bowing technique were rather similarly performed by the participants. The section provided a consistent segment for comparison across participants and minimized potential variability arising from potential differences in musical interpretation, phrasing, or subsequent movements. The starting point was set at the first bow stroke (upstroke at the upbeat) and ended at the beginning of the third measure. The synchronized recordings of the motion capture and the EMG of each player were resampled to an equal length of this section to avoid tempo variances.

The motion analysis and the post-processing of the EMG data have been performed using Matlab version R2024a (MathWorks Inc., Natick, Massachusetts). Statistical analyses were conducted with SPSS 31 (Armonk, NY: IBM Corp.). Descriptive statistics report the mean and the standard deviation (SD). Comparisons of EMG values between muscles used *t*-test with paired samples. Analyses of variances across the three measuring points were performed with repeated measure tests reporting the *F*-value and the *p*-value of the linear within-subject contrast as well as the partial Eta-Squared as effects size (p.η^2^). As Cohen (Cohen, 1988) classified large effects at >0.14, in this study due to the sample size, large effects were considered at >0.20. On significance, single t-test comparisons were calculated between measurement points. Post-Hoc analyses used the Tuckey-HSD correction. The level of significance was set to *p* = 0.05.

## 3 Results

Table 1 shows all mean EMG values across the 14 participants of each muscle group. In the forearm flexors, the mean dynamic EMG values of the left flexors were significantly higher than of the right flexors (t > 7.3, *p* < 0.001). In the baseline condition at rest (note the reduced sample n = 10 due to technical issues), there were no significant differences between right and left flexors. In the same line, the mean dynamic EMG values of the left biceps were significantly higher than of the right biceps (t > 6.9, *p* < 0.001) where no significant differences were found in the baseline condition. In contrast, for the upper trapezius, the mean dynamic EMG values of the right trapezius were significantly higher than of the left trapezius (*t* > 1.9, *p* < 0.036). Still, the baseline EMG values were not significantly different between both sides.

**Table 1:** Mean values with standard deviation (SD) of the dynamic EMG values of each muscle group including the maximum value (Max) and the peak dynamic method value (PDM) at the different measurement times (T).

Measures	T1		T2		T3		Statistics		
	Mean	SD	Mean	SD	Mean	SD	*F*-value	*p*-value	p.η^2^
Flexors right (in μV)	18.3	9.6	16.8	10.1	16.4	9.1	5.2	0.040	0.29
Max (in μV)	43.1	22.4	45.1	20.9	40.9	18.3	<1	n.s.	0.03
PDM (in %)	**45.8**		**40.9**		**41.9**		**5.3**	0.**039**	0.**29**
Baseline condition (in μV)	4.3	0.9	4.7	1.7	4.2	0.6	<1	n.s.	0.02
Flexors left (in μV)	46.5	22.8	43.2	21.1	41.9	20.3	16.3	0.001	0.56
Max (in μV)	**107.4**	**36.1**	**96.6**	**31.5**	**96.3**	**36.3**	**6.7**	0.**022**	0.**34**
PDM (in %)	44.8		45.3		44.9		<1	n.s.	<0.01
Baseline condition (in μV)	4.6	3.1	5.8	2.7	4.6	2.2	<1	n.s.	<0.01
Biceps right (in μV)	30.8	14.7	31.0	15.3	29.0	14.1	14.7	0.002	0.53
Max (in μV)	67.5	32.9	69.2	37.7	63.4	32.1	2.1	n.s.	0.14
PDM (in %)	45.5		45.4		44.2		<1	n.s.	0.03
Baseline condition (in μV)	5.2	1.3	4.9	0.9	5.0	1.6	<1	n.s.	0.01
Biceps left (in μV)	95.2	38.5	88.5	38.5	87.5	33.9	7.1	0.020	0.35
Max (in μV)	204.4	130.7	189.1	111.5	181.4	98.5	3.2	n.s.	0.19
PDM (in %)	51.1		49.9		50.8		<1	n.s.	<0.01
Baseline condition (in μV)	4.1	1.1	5.0	2.3	4.2	1.7	<1	n.s.	<0.01
Trapezius right (in μV)	53.9	24.1	50.9	24.9	44.6	22.0	14.1	0.002	0.52
Max (in μV)	**111.6**	**57.0**	**111.5**	**56.3**	**96.9**	**49.1**	**9.2**	0.**010**	0.**42**
PDM (in %)	49.2		46.3		48.2		<1	n.s.	0.02
Baseline condition (in μV)	**9.5**	**3.3**	**8.8**	**4.5**	**7.2**	**2.9**	**53.0**	**<0.001**	0.**86**
Trapezius left (in μV)	38.2	17.7	37.6	17.4	31.3	14.9	5.1	0.041	0.28
Max (in μV)	**80.6**	**53.5**	**80.7**	**48.9**	**67.4**	**49.2**	**6.2**	0.**027**	0.**32**
PDM (in %)	49.6		47.5		50.0		<1	n.s.	<0.01
Baseline condition (in μV)	**10.1**	**2.9**	**9.7**	**4.2**	**7.1**	**1.3**	**19.9**	0.**002**	0.**69**
Right Elbow angle right (in °)	84.0	14.0	83.9	13.9	83.8	14.0	<1	n.s.	<0.01
Right Elbow motion range (no unit)	3.73	1.49	3.59	1.36	3.99	1.89	1.2	n.s.	0.08
Right Wrist motion range (no unit)	6.15	2.25	6.19	2.14	6.54	2.55	<1	n.s.	0.06

The baseline condition reports the RMS values at rest. For the motion, the mean right elbow angle as well as the elbow and wrist 3D motion ranges are shown. Statistics of the repeated measure analysis across the measuring times report the *F*-value and the *p*-value (p. η^2^: partial Eta-Squared; n.s., not significant; in bold: significant effects).

The repeated measure analysis found that in both forearm flexors the mean EMG values reduced significantly over the measurements. In the both flexor muscles, there was a significant decrease between the first and second (control condition) measurements (right: *p* = 0.044, left: 0.006) which did not decrease significantly further between the second and third (intervention condition) measurements.

For the biceps muscles, the mean EMG values of the right biceps did not reduce significantly between the first and second measurements, but there was a significant decrease over the intervention (*p* = 0.023). Thus, the left biceps muscle activity showed no significant reduction between first and second measurements (*p* = 0.065) or between the second and third measurements, but decreased significantly between the first and third measurements (*p* = 0.01).

Both trapezius muscle activities showed a significant reduction in their EMG values across the measurements. In the right trapezius, the muscle tone decreased continuously over the measurements (T1-T2: *p* = 0.003; T2-T3: *p* = 0.005). The mean muscle activity in the left trapezius did not reduce significantly between the first and second measurements, but decreased significantly between the second and third measurements (*p* = 0.043).

For the maximum dynamic muscle activity, there were significant reductions in the left flexors and both trapezius muscles. Similar to the mean EMG values, there was a significant decrease in the left flexor muscles between the first and second measurements (*p* = 0.012), but not between the second and third measurements. In this both trapezius muscles, the maximum values did not decrease significantly between the first and second measurements, but reduced significantly between the second and third measurement (right: *p* = 0.004, left: 0.039).

The normalized PDM values represent the level of muscle activity in relation to the maximum dynamic muscle activity during the performance. In all muscle groups, the mean PDM was between 40 and 51%. Significant changes across the three measurements were only found in the right flexor muscles with a significant decrease between the first and second measurement (*p* = 0.03), but without further reductions over the intervention.

In both trapezius muscles, there were significant decreases in the mean muscle activity of the static baseline measurement. Furthermore, the mean muscle activities were not significantly different between the first and the second measurements, but over the intervention, the muscle activities reduced significantly on both sides (right: *p* = 0.019, left: *p* = 0.023).

There were no significant changes in the mean right elbow angle or the mean motion ranges in the elbow or in the wrist.

Across genders, no significant differences were found in the mean EMG values. However, there was a significant difference in the mean elbow angle between genders (T1: *p* = 0.004, T2: *p* = 0.023, T3: *p* = 0.033) with larger angles for the male musicians (about 97°) compared to the female musicians (about 73°). Nevertheless, there was no significant difference in the movement ranges in the arm and the wrist movements across genders.

Considering the reported musculoskeletal complaints, the EMG values were analyzed using the repeated measures analysis with the stated region of problems as factor such as shoulder and/or neck region, arm and/or wrist region, and no complaints. The analyses revealed no significant main effects or interactions in the mean EMG values and the maximum EMG values. In the PDM values, a significant interaction effect was found for the right biceps (*F*(2,11) = 4.5, *p* = 0.036) where the players with shoulder/neck complaints increased in the PDM value while the others decreased the PDM value. There was also a significant difference in the mean right trapezius PDM values between the groups (*F*(2,11) = 5.0, *p* = 0.028) with higher PDM values in the group with shoulder and neck complaints and the group without complaints compared to the group with arm and wrist complaints. In the right elbow motion range, the players with arm and wrist complaints were significantly higher than the other two groups (*F*(2,11) = 6.3, *p* = 0.015).

### 3.1 Playing positions

The explorative Post-Hoc analysis of the playing positions revealed four different playing types. The playing types were classified for the height of the violin (high/low) and the angle of the violin in rotation to the frontal plane (about 20°/ about 45°). The motion capture visualisation showed that six players hold the violin at an angle of about 20° (+/− 5°) to the frontal plane. Considering the height, four of them hold the violin at about shoulder level (high position) and two at about breast level /low position). The remaining eight players all held their violins at an angle of around 45° (+/− 5°). Half of them held it at shoulder level and the other half held it at breast level (Table 2). No significant distribution difference in the position types were found between genders.

**Table 2:** Classification of four position types regarding the height of the violin (high/low) and the angle of the violin (~ 20°/~45° to the frontal plane) with the number of players and the mean EMG values with standard deviation (SD) of the muscle groups in the three measurements.

Position		High				Low			
Angle		EMG in μV (SD)				EMG in μV (SD)			
		*N*	T1	T2	T3	*N*	T1	T2	T3
20°	Flexors right	4	19.5 (11.1)	16.8 (9.5)	15.6 (9.4)	2	19.9 (5.5)	17.9 (4.5)	16.6 (6.5)
	Flexors left		47.1 (8.7)	42.2 (8.8)	42.0 (10.9)		51.1 (16.3)	49.4 (17.1)	41.2 (14.3)
	Biceps right		19.8 (5.7)	18.5 (5.2)	17.1 (5.5)		45.5 (5.9)	46.1 (10.3)	40.9 (4.3)
	Biceps left		65.1 (31.9)	64.9 (30.6)	57.0 (23.3)		125.4 (29.7)	110.9 (33.3)	113.9 (29.2)
	Trapezius right		31.8 (14.4)	31.2 (12.8)	28.5 (12.6)		97.7 (30.2)	94.3 (28.4)	72.6 (23.7)
	Trapezius left		26.5 (17.2)	22.8 (15.4)	19.8 (17.3)		38.7 (15.6)	39.1 (25.8)	32.8 (5.9)
45°	Flexors right	4	19.0 (5.8)	18.8 (3.9)	18.2 (6.1)	4	14.6 (4.2)	14.3 (5.1)	15.1 (5.9)
	Flexors left		39.3 (15.3)	35.2 (13.7)	35.7 (14.7)		50.7 (8.7)	49.3 (14.1)	48.4 (11.6)
	Biceps right		24.7 (7.2)	25.7 (6.9)	23.1 (7.2)		41.2 (29.5)	41.3 (33.0)	40.8 (30.5)
	Biceps left		68.8 (19.3)	68.9 (23.7)	66.4 (26.3)		136.4 (34.7)	120.4 (45.4)	125.7 (33.9)
	Trapezius right		43.8 (24.1)	38.9 (22.5)	34.5 (17.9)		64.1 (28.7)	60.9 (26.9)	56.7 (23.5)
	Trapezius left		26.7 (20.6)	29.4 (24.3)	25.4 (19.2)		61.1 (35.7)	59.8 (33.9)	48.5 (32.8)

Across the performances, the players did not change the position of the violin between the three measurements. All players performed three times with the violin at the same height and angle, and stayed within the same playing type. The analyses of these playing types were difficult due to the small sample size in each type and were limited in statistical power. They should therefore be interpreted with caution. Thus, a repeated-measures analysis was conducted with two different factors: (1) including all four types, and (2) including two types, in which the high and low playing positions were combined into separate groups of high and low position. For the first factor, the numbers of participants in the groups were very small. To obtain more reliable analyses, the second factor was chosen to perform analyses with more participants in each group.

The analysis of the mean EMG muscle activity with all four position types as factor (see Table 2) showed significant effects in the left biceps (*F*(3,10) = 4.5, *p* = 0.03, p.η^2^ = 0.58) and the right trapezius (*F*(3,10) = 3.8, *p* = 0.048, p.η^2^ = 0.53). In the left biceps, there was a significant difference between the two high and the two low position types (Post-Hoc, *p* = 0.047) with higher values in the two low position types. The muscle activity of the right trapezius in the 20° high position type was significantly lower than in the 20° low position type (Post-Hoc, *p* = 0.05).

Considering the analysis across the three measurements with the factor of all four position types, significant effects were found in all muscle groups similar to the findings mentioned above without the factor included. Significant interaction effects were found in the right biceps (*F*(3,10) = 4.1, *p* = 0.040, p.η^2^ = 0.55) and in the right trapezius muscle (*F*(3,10) = 4.7, *p* = 0.027, p.η^2^ = 0.59). In both muscle regions the activity reduced most in the 20° low position type.

In the wrist movement range, there was a significant interaction effect (*F*(3,10) = 5.1, *p* = 0.021, p.η^2^ = 0.61) with large reductions of the motion in the 20° low position type and increases in the 20° high position type.

In the maximum dynamic muscle activity, there were significant interaction effects in the right biceps (*F*(3,10) = 4.3, *p* = 0.034, p.η^2^ = 0.56), and the right trapezius (*F*(3,10) = 5.6, *p* = 0.017, p.η^2^ = 0.63) with the largest decreases over the intervention in the 20° low position type.

No significant interaction effects were found in the PDM values across the measurements in the four position types.

The repeated measure analyses with the factor of two groups with high and low playing position found no significant interaction effects in the mean EMG values and in the maximum muscle activity. Between the high and low position groups, significant effects were found in the mean values of the left biceps muscle (*F*(1,12) = 12.2, *p* = 0.004, p.η^2^ = 0.50) and the left trapezius (*F*(1,12) = 8.7, *p* = 0.012, p.η^2^ = 0.42) with higher values in the low position group.

For the mean PDM values considering the two position groups, a significant interaction effect was found in the right biceps muscle (*F*(1,12) = 7.6, *p* = 0.018, p.η^2^ = 0.39) with the high position group lowering the PDM value from 46 to 40% whereas the low group increased the PDM value from 46 to 50% over the three measurements.

Regarding the musculoskeletal complaints, there was a significant distribution difference between the two position groups (χ^2^ = 7.5, *p* = 0.023, exact: χ^2^ = 6.9, *p* = 0.05). In the high position group, three players had no complaints, four players reported arm and/or wrist problems and one player had shoulder and/or neck problems. In the low position group, only one player had no complaints, five players reported shoulder and/or neck problems and none had arm and/or wrist problems.

## 4 Discussion

This study investigated the effects of a brief body-oriented intervention on muscle activity and movement patterns in violinists. Participants performed the opening section of Bach’s *Partita in D minor* while biomechanical and physiological measurements were recorded. The assessments included motion analysis of the right upper limb, evaluation of the violin playing position, and electromyographic (EMG) recordings of arm and shoulder muscles on both sides. In addition, the players were asked about any existing playing-related health problems, particularly in the shoulder, neck, arm and wrist regions.

### 4.1 General effects

In accordance with the literature (Steinmetz et al., 2015; Rensing et al., 2018), approximately three-quarters of the players reported health problems that interfered with their playing. The shoulder region was the most frequently mentioned area, which is consistent with the region where violinists most often experience complaints (McCrary et al., 2016). That is still a very large number of players with certain health issues, which should continue to be taken into consideration. The main goals of this study were to investigate potential relationships between self-reported health problems and body regions of increased muscular load, to evaluate whether a brief intervention could moderate muscular fatigue and to examine possible differences in these aspects between playing positions.

To assess muscle activity, the raw EMG signals were filtered with standard methods and the mean EMG value was calculated. As the mean EMG values were not normalized, the analyses were primarily explorative and descriptive and should be regarded as an indication of general muscle activity levels. Moreover, the static baseline recordings indicated no unusually high resting muscle activation or apparent measurement artefacts and showed to be very consistent across players and muscle groups (note the reduced sample in this condition). This made it possible to draw some conclusions from the comparisons of the EMG values between players.

The analyses of the general EMG mean muscle activity revealed that the flexor muscles and biceps on the left arm were more active than on the right arm, whereas the right upper trapezius muscle was more active than the left trapezius. These findings confirm results of previous research of higher muscle activities in the left arm muscles in violinists (Mann et al., 2021). During violin performance, the left arm remains comparatively static, primarily supporting the instrument while the fingers execute note production on the fingerboard (Rensing et al., 2018; Schemmann et al., 2018). Therefore, increased flexor muscle activity in the left arm is mainly associated with finger movements and the force applied to the strings. Higher activation of the left biceps reflects a dual role in holding the instrument while also facilitating finger movements and position shifts along the fingerboard. The left trapezius muscle is often tensed when the left shoulder is raised to hold the violin in place between the shoulder and the chin. In contrast, the right trapezius is actively involved in bowing movements, assisting in shoulder elevation and rotation of the right arm to position the bow appropriately for the string being played (Mann et al., 2023). The results of the present study support that asymmetric muscle activity and high muscle tensions in individual muscle groups are found.

In contrast to other studies, the present study found no significant gender-related differences in either EMG or PDM values. In a previous study, Mann and colleagues (Mann et al., 2021) reported higher muscle activity in female violinists compared with male violinists. The authors attributed these differences to gender-related anatomical and physiological characteristics, suggesting that shorter arm and finger lengths in female participants resulted in greater muscle activation during violin performance. Therefore, the reported gender-specific differences in muscle activity could not be confirmed with the measures used in this study.

### 4.2 Effects across the measurements

For analyzing the effects across the measurements, repeated-measures analyses were conducted accounting for within-subject changes. With this, even small changes in the mean EMG values were found to have statistically significant effects. This was especially the case for the right biceps muscle. Examination of the individual EMG data of each player (see Supplementary material) indicates that most participants showed a steady reduction in the right biceps muscle activity across the measurements. This general within-subject trend explained the significant main effect.

Overall, the muscle activity in all muscle groups decreased significantly across the three measurements. However, the timing of these reductions differed between muscles. Activities in some muscle groups declined during the break but showed no further reduction over the intervention. In contrast, activities in other muscle groups decreased only after the intervention. These findings suggest the presence of two distinct effects: (1) the break itself promoted relaxation in a subset of muscles, whereas (2) the intervention elicited additional reductions in muscle activity in muscles that did not reduce significantly during the pause.

Over the break, the forearm flexor muscles on both sides and the left biceps decreased in muscle activity. It is possible that these muscles were more active before the first performance, may be due to the unusual situation. During the break, the participants may have become accustomed to the situation and found the pause relaxing, which was reflected in a decrease in muscle activity. The muscle activity during the second performance was then at a lower level of muscle tension. Under these conditions, it may not have been possible – nor would it have been appropriate – to achieve a further reduction in muscle activity through the intervention.

The higher muscle tension observed in the first measurement may have been an arousal response to the unfamiliar situation, and the reduced muscle tension in the second measurement as the result of relaxation during the break. The differences in the third measurement might have been caused by the additional relaxing situation of the intervention. However, since some muscles did not change during the first break, it was not to be expected that this would have been changed with another break. The effects of the specific relaxations of certain muscles were therefore evident for been influenced by the intervention. Here, an effect of Functional Integration (FI) can be interpreted, which, through movement impulses, stimulates the brain to create new patterns of activity in the interaction of the muscles involved (Brummer et al., 2018; Berland et al., 2022). The results suggest that these deeply ingrained movement patterns in violin playing can only be influenced by new impulses, such as those conceptually pursued by FI. To verify this hypothesis, a comparative study between different interventions would need to be carried out. In such a study, warm-up and relaxation techniques that the musicians can perform themselves could be compared with professional guidance provided through a brief Feldenkrais intervention.

A standard normalization of the EMG values has been performed using the Peak Dynamic Method (PDM), which expresses the mean EMG activity as a percentage of the maximum EMG activity during the performance. Across all muscle groups, PDM values were approximately 50% or slightly lower, indicating that average muscle activation during performance was about half of the peak dynamic activation. Although the maximum EMG values changed across the measurements, the normalized PDM values remained stable. This suggests that, despite changes in the maximum dynamic activity, the players consistently performed at a similar relative level of muscle activation with respect to their maximum dynamic activity.

The baseline measurements were used to assess muscle activity during quiet standing without the instrument. Unfortunately, the sample was reduced due to technical problems. However, the results showed that the flexor muscles and the biceps muscles on both sides had rather low static activation levels, which did not change significantly across the measurements.

The transformation matrix was used to analyze movement patterns during performance. Following an approach similar to the biomechanical model proposed by Visentin and Shan (Visentin and Shan, 2003), movements of specific body segments were reconstructed from the IMU data after normalizing segment lengths to unity. This procedure enabled comparisons across participants while minimizing the influence of individual differences in body size. The results revealed no significant changes in the motion ranges of the right arm across the three measurements, indicating that the overall movement patterns remained consistent throughout the measurements.

Regarding the elbow angle of the right (bowing) arm, male violinists showed a greater mean elbow angle than female violinists. This difference may be attributable to physiological factors, such as larger arm length in male players. However, no gender-related differences were observed in the EMG activity, suggesting that the variation in elbow angle did not influence muscle activation. Furthermore, as the mean elbow motion range also did not differ between genders, both groups appear to have performed with similar bowing patterns, differing primarily in their initial elbow angle. Since arm length was not assessed in this study, future research should include anatomical variables, along with overall body size, to better account for potential biomechanical differences.

### 4.3 Intervention effects

A substantial intervention effect was observed in the upper trapezius muscle activation on both sides, especially of the left trapezius muscle that is commonly involved in playing-related musculoskeletal complaints among violinists (Rensing et al., 2018). The muscle activity decreased following the intervention, indicating a reduction in muscle tension. This effect was observed in both the static baseline activity and the dynamic playing activity. The trapezius muscles displayed generally higher baseline mean EMG values than the other muscle groups, which suggests that there was already more shoulder muscle tension at the beginning. The observed decrease over the intervention in the baseline activity of the trapezius muscles is consistent with the reduction in the dynamic activation. These findings suggest that the participants adopted more relaxed shoulder activity, which they were able to transfer to their performance. When playing the violin, the left upper trapezius muscle needs a certain activity level required for the performance. The present findings suggest that this activation level can even be further reduced through specific exercises, such as those implemented in the intervention. As there was no reduction in trapezius activity during the pause, it seems that only the intervention promoted the muscular relaxation.

Another finding regarding the effects of the intervention was that the right biceps and the right trapezius muscle showed a clear reduction in muscle activity after the intervention. Both muscles are involved in the bowing movements of the right arm. The results indicate for a more relaxed movement of the bow after the intervention. In addition, the motion ranges of the right elbow and wrist as well as the elbow angle remained unchanged across the measurements. These findings suggest that the players maintained a similar bowing kinematic while playing with less unnecessary muscle activation. A possible explanation is that the mechanical demands of the bowing movement were more evenly distributed across the muscle chain of the right arm and the shoulder after the intervention, reducing the load on individual muscles and resulting in more relaxed and probably efficient bowing movements.

An additional indicator of muscle load was the maximum dynamic muscle activity occurring during the performance. This measure showed substantial inter-individual variability, with the standard deviation often reaching about half of the mean value. Despite this variability, significant changes across the measurements were observed for the left forearm flexors and both trapezius muscles. While the maximum activity of the left flexors decreased after the short break, the maximum activity of the trapezius muscles only decreased significantly after the intervention.

In summary, the findings suggest that the players were able to perform the musical piece using the same movement pattern while requiring less muscle activation. Although a brief pause before the second playing was sufficient to reduce activation levels in some muscles, the intervention was required to achieve significant reductions in activity levels particularly in those muscle groups that are often tense when playing the violin. Experiencing violin playing with reduced muscle activation may help musicians develop a perception of an efficient and relaxed playing. This experience may enable players to recognize excessive muscle tension in other performance situations and consciously adjust their muscle activation, thereby reducing the risk of muscle fatigue.

### 4.4 Playing positions

Violin playing is a highly individualized motor task that is influenced by multiple interacting factors which violinists tend to adopt for particular playing positions. Therefore, it is recommended that the potential link between different playing behaviors and muscle activity is considered (Mann et al., 2023). Hildebrandt and colleagues (Hildebrandt et al., 2021) investigated muscle activity across four different violin playing positions while playing notes with the left hand and with the right hand resting alongside the body rather than performing bowing movements. To standardize the playing positions, a custom apparatus was used to hold the violin in place. The four conditions were defined by two angles of the violin relative to the frontal plane (20° and 50°) and two inclination angles (20° and 50°). However, these experimentally imposed positions may not have reflected the violinist’s habitual playing position. Consequently, performing in unfamiliar positions could have caused unnatural muscle activations. Therefore, in the present study, participants were instructed to play in their usual manner, and their playing was analyzed retrospectively to identify specific playing positions.

According to Hildebrandt and colleagues (Hildebrandt et al., 2021), in the present study, two different angles of the violin relative to the frontal plane were found with similar angles of 20° and 45°. By contrast, the height of the violin relative to the shoulders was found to be a key factor. Almost half of the violinists held their instrument at shoulder level, while the other half held it with the scroll approximately at breast level. These are rather common playing positions among violinists, and they often depend on how the musician was taught to play the violin or following a certain style in connection to the literature.

Nevertheless, the inclination of the violin may represent an additional factor influencing playing. However, the violin’s tilting is inherently coupled to the position of the bowing arm. In the high playing position with 20° angle to the frontal plane, a greater violin inclination is required to facilitate efficient bowing movements of the right arm. A smaller inclination would demand an unusual elevation of the right arm to maintain proper bow contact across all strings. This would result in higher muscle activity as has been found by Hildebrandt and colleagues (Hildebrandt et al., 2021). On the other hand, in the lower playing position, a smaller violin inclination better supports the natural movement of the bowing arm. For these reasons, violin inclination was not included as a factor in the present study.

Despite the small sample sizes in the playing types, analyses of muscle activity and playing motions were performed. This followed an exploratory approach, and the findings should be considered in light of the sample sizes. By analyzing the muscle activity across the four playing positions, the results indicated position-dependent differences in some muscle activity. Specifically, the left biceps showed higher mean muscle activity when the violin was held in the low playing position compared with the shoulder-level position. This may be explained by the increased demand placed on the arm to support the weight of the instrument in the low position. In contrast, when the violin is held at shoulder level, the shoulder musculature may contribute to supporting the instrument, thereby reducing the activity on the biceps.

The right upper trapezius muscle activity was found to be highest in the 20° low playing position and lowest in the 20° high playing position. An explanation could be that this muscle supports the bowing movements of the right arm. When the violin is held at a low height almost directly to the front, it might be that the right arm has to move over the instrument to bow the upper stings. When hold at a higher level with an appropriate inclination of the violin, the bowing arm needs less lifting to reach the upper strings.

In Hildebrandt and colleagues (Hildebrandt et al., 2021), the highest muscle activity was found when the violin was held 20° angle to the frontal plane and with the small inclination of 20° in the violin. This particular position forces the left arm to perform with an unusual inner rotation that can cause higher muscle tension. The present study found no difference in the activity of the left forearm flexors between the four playing positions. This suggests that either the players performed with an optimal inclination angle of the violin to minimize uneconomical rotations and muscle tension, or they were accustomed to this specific position.

As the muscle activity varied depending on the violin playing position, it could be assumed that the intervention led to an altered playing position, which subsequently reduced muscle activity. Nevertheless, the analysis found no evidence that the players changed their playing position across the measurements. This supports the interpretation that the reduction in muscle activity observed can be attributed to the effects of the intervention.

Across the measurements, EMG activity of the right biceps and right trapezius muscles decreased after the intervention in the 20° low playing position. This finding suggests that players adopting this playing position may particularly benefit from the intervention. Notably, these two muscle groups exhibited comparatively high mean EMG activity relative to the other playing position types. In contrast, the pause condition did not result in a considerable activity reduction in these muscles. These findings highlight the potential value of the intervention in players performing in a low playing position. However, the results should be interpreted with caution, as this playing position type consisted of only two players. Further research with a larger sample size is required to confirm these preliminary findings and to better understand the effects of the intervention in this specific playing position.

### 4.5 Correlations with self-reported musculoskeletal pain

Another factor that has been extensively investigated is the presence of musculoskeletal problems in relation to muscle activity. Several studies have reported higher EMG activity in violinists with musculoskeletal complaints compared to those without complaints (Park et al., 2012; Kok et al., 2018; Möller et al., 2018; Schemmann et al., 2018). In the present study, self-reported musculoskeletal complaints were assessed and categorized into three groups: (1) shoulder and/or neck complaints, (2) arm and/or wrist complaints, and (3) no complaints. Contrary to previous findings, general differences in the mean EMG muscle activity between these groups could not be confirmed. However, muscle-specific differences in the PDM values were observed for the right biceps and the right trapezius across the measurements.

Players reporting shoulder and/or neck complaints showed higher PDM values in the right trapezius than those in the other groups. Overall, their mean trapezius activity was closer to their maximal activation throughout each measurement, which may reflect a higher muscle tension associated with experienced pain or protective muscle behavior. Interestingly, the PDM values of the right biceps in this group increased after the intervention. One possible explanation is that the intervention improved participants’ awareness of shoulder muscle activation, leading them to compensate by relying more on arm movements during playing, thereby increasing activation of the biceps. However, this interpretation remains speculative and should be investigated in future studies.

Regarding the playing positions, a significant difference in the distribution of musculoskeletal complaints was observed between players using low and high playing positions. Among players in the low playing position, the majority (5 of 6) reported shoulder and/or neck complaints. This group also exhibited higher activation of the left biceps and the left upper trapezius muscles compared with players using the high playing position. These findings suggest that the low playing position may place greater mechanical demands on the left shoulder and cervical region. One possible explanation is that this playing position promotes a slightly forward-flexed trunk and head posture, potentially adopted to improve the view to the music stand (Spahn et al., 2014). Future studies should investigate whether this playing position contributes to the observed increase in shoulder and neck complaints. In contrast, players using the high playing position more frequently reported complaints affecting the arm and/or wrist (4 of 8). It seems that they have different demands when it comes to playing.

### 4.6 Limitations

A major limitation of the present study was the relatively small sample size. Although a total of 14 violinists participated, the number of participants within each playing position subgroup was substantially reduced, limiting the statistical power of between-group analyses. For example, analyzing the muscle activity across the four playing positions substantially reduced the group sizes for comparisons. Consequently, a descriptive approach was adopted to explore potential differences between playing positions, and the findings should be interpreted with careful consideration. Future studies should aim to recruit a larger number of violinists in each playing position type to enable more robust and reliable comparisons.

In this study, it was not possible to extract the tilting angle of the violin from the recorded data. It might be possible that the tilting of the violin can be a third factor relating to the playing position that needs to be considered when investigating health issues in violinists. Investigating the influence of playing positions on muscle activity with all three factors would require a much larger sample size.

For normalization of the EMG data, the Peak Dynamic Method (PDM) was applied. This method is particularly appropriate for comparing muscle activation patterns during dynamic motor tasks, such as walking, running, or cycling, because it normalizes EMG amplitudes to the peak activation observed during the task itself. In contrast, the standard normalization of Maximum Voluntary Contraction (MVC) is more suitable when the objective is to quantify muscle activation relative to an individual’s maximal voluntary force capacity. However, future studies should consider performing MVC normalization, particularly to facilitate comparisons with previous studies.

Nevertheless, performing MVC measurements immediately before a performance of a complex and technically demanding musical piece may influence the performance. Specifically, the high level of muscle activation required during MVC testing could induce temporary fatigue, potentially impairing the musical performance. Conversely, musicians may not apply a true maximal contraction during the MVC procedure if they anticipate performing the piece immediately afterwards to avoid potential fatigue. Whether these factors really affect MVC validity or subsequent performance should be investigated in future studies, particularly in professional musicians. It could also be considered to conduct MVC measurements at the end of the data collection process, in order to avoid fatigue issues prior to playing. This may be a possible alternative for less physically demanding tasks to avoid influencing the MVC due to muscle exhaustion. This should also be investigated in future studies.

Despite the specific positioning of the violin on the left shoulder, relatively few studies have examined the role of ergonomic devices, such as shoulder and chin rests, in relation to playing posture and musculoskeletal disorders. A systematic review by Chi et al. (2020) concluded that the use of shoulder rests influences muscle activity. However, the available evidence remains insufficient to determine their overall physical impact on violinists, highlighting the need for further research. In the present study, the use of shoulder or chin rests was not controlled or analyzed. Players performed under their usual playing conditions, with no modifications to their playing technique or ergonomic setup across the measurements. Consequently, the observed changes cannot be attributed to the use of any ergonomic device. Future studies should investigate the effects of such devices and their interaction with the playing position to better understand their potential role in reducing musculoskeletal complaints.

Regarding a counterbalance of intervention and control condition, no randomization of the order was performed due to a possible retention effect of the intervention. A break condition before the intervention is not comparable to a break condition after the intervention. One could have performed a specific control condition setting, where players had to come on an extra date for been measured before and after a break. However, other methodological issues would have arisen, such as problems of day and mood related muscular differences as well as differences in the measurements due to changes in the sensor positions. Thus, a setting was used, where no sensor replacement was necessary. If possible, future studies investigating effects of interventions should consider using a specific control condition.

Finally, no audio recordings of the performances were made in this study. Therefore, it was not possible to compare the performances across measurements with respect to sound dynamics, intensity, or overall performance quality. It is possible that performing the same musical piece with reduced muscle tension may lead to subtle variations in acoustic characteristics. Future studies should therefore include audio recordings of the performances to enable analyses of both biomechanical and acoustic aspects of musical performance.

## 5 Conclusion

The results, which showed a reduction in muscle activity across all measurements, made it clear that muscle tension while playing the violin is sensitive to changes in conditions, even when performing a piece is not significantly impaired. These findings suggest that muscle activity should be given greater consideration in violin playing, as comparable levels of performance may be achieved with lower and more evenly distributed muscle activation. Therefore, training approaches that promote greater awareness of individual muscle use may be beneficial for developing more efficient patterns of muscular activation during violin performance.

In the present study, the Feldenkrais Method was employed as the body-oriented intervention. The results indicate that this intervention had specific beneficial effects on the muscular activity. However, it remains possible that other body-oriented approaches, such as the Alexander Technique, Dispokinesis, yoga, or relaxation- and meditation-based methods, may produce comparable effects. More broadly, body-oriented interventions may contribute to both physiological and psychological improvements, although the extent and nature of these effects may vary depending on the specific method employed.

Given these potential benefits, the integration of evidence-based body-oriented programs into music training curricula should be employed (Spahn et al., 2017). Such programs can contribute to the prevention of long-term musculoskeletal problems while simultaneously promoting health literacy and greater awareness of healthy movement and bodily practices among musicians.

## Data Availability

The raw data supporting the conclusions of this article will be made available by the authors, without undue reservation.
