# Supplementary material for: Effectiveness of a brief body-oriented intervention on muscle activities when playing the violin
Source: Front Psychol. 2026 Oct 14;17:1944291. doi: 10.3389/fpsyg.2026.1944291 (PMC13713406; doi:10.3389/fpsyg.2026.1944291)
Supplement: Supplementary file 1 [file Table_1.DOCX]

Supplementary Material

Effectiveness of a brief body-oriented intervention on muscle activities when playing the violin

# 1 Complete Table

| **ID** | **Time** | **Flex r** | | **Flex l** | | **Biz r** | | **Biz l** | | **Trap r** | | **Trap l** | | **Elbow r** | **3D Range** | |
| --- | --- | --- | --- | --- | --- | --- | --- | --- | --- | --- | --- | --- | --- | --- | --- | --- |
|  |  | **M (SD)** | **Max**  **PD%** | **M (SD)** | **Max**  **PD%** | **M (SD)** | **Max**  **PD%** | **M (SD)** | **Max**  **PD%** | **M (SD)** | **Max**  **PD%** | **M (SD)** | **Max**  **PD%** | **Angle**  **M (SD)** | **Elbow r SD** | **Wrist r SD** |
| P1 | T1 | 14.1 (6.09) | 28.3 49.8 | 21.2 (15.2) | 57.7 36.8 | 18.4 (13.2) | 55.8 33.0 | 71.0 (15.9) | 103.5 68.6 | 77.2 (36.8) | 152.1 50.2 | 54.2 (24.6) | 102.9 52.7 | 71.9 (16.2) | 7.17 | 11.13 |
|  | T2 | 17.4 (19.4) | 83.5 20.8 | 18.2 (13.4) | 50.7 35.9 | 22.6 (28.8) | 109.8 20.6 | 78.7 (31.7) | 186.2 42.3 | 69.0 (35.9) | 130.8 52.8 | 62.7 (28.4) | 121.4 51.7 | 72.1 (17.3) | 5.31 | 8.78 |
|  | T3 | 12.9 (9.3) | 39.9 31.4 | 18.5 (13.0) | 49.5 37.3 | 14.9 (12.5) | 55.6 26.9 | 85.4 (31.3) | 173.7 49.2 | 59.7 (34.6) | 128.8 46.4 | 50.4 (17.4) | 81.4 61.9 | 69.2 (18.6) | 6.83 | 10.32 |
| P2 | T1 | 9.3 (3.5) | 16.4 56.6 | 44.5 (23.6) | 109.5 40.6 | 15.0 (11.1) | 53.0 28.3 | 149.4 (70.7) | 353.8 42.2 | 55.4 (23.9) | 110.4 50.1 | 33.5 (25.5) | 97.6 34.3 | 75.7 (7.9) | 2.83 | 4.69 |
|  | T2 | 8.4 (4.6) | 20.1 41.8 | 39.3 (25.5) | 104.0 37.8 | 12.1 (6.9) | 33.1 36.5 | 103.7 (57.6) | 224.0 46.3 | 55.7 (36.8) | 139.8 39.8 | 33.0 (30.1) | 97.5 33.9 | 77.6 (8.3) | 2.12 | 4.53 |
|  | T3 | 8.8 (3.6) | 18.4 48.1 | 41.3 (17.2) | 76.9 53.7 | 13.1 (7.8) | 33.8 38.7 | 138.1 47.1 | 266.4 51.8 | 52.0 (23.5) | 109.2 47.6 | 22.2 (15.8) | 67.7 32.8 | 72.6 (8.42) | 2.96 | 6.04 |
| P3 | T1 | 31.3 (15.4) | 69.7 44.8 | 44.9 (13.9) | 74.6 60.1 | 17.7 (11.8) | 52.9 33.5 | 55.8 (19.8) | 96.2 58.1 | 18.3 (15.2) | 60.4 30.3 | 8.3 (2.2) | 13.7 60.8 | 119.5 (17.9) | 6.17 | 8.47 |
|  | T2 | 28.6 (15.4) | 60.5 47.2 | 40.6 (10.0) | 83.6 48.5 | 15.9 (9.8) | 44.3 35.9 | 55.6 (22.4) | 110.7 50.2 | 21.7 (21.5) | 82.5 26.4 | 7.3 (1.9) | 12.8 56.9 | 102.1 (23.3) | 5.97 | 10.9 |
|  | T3 | 27.7 (13.6) | 58.6 47.3 | 39.8 (13.2) | 73.9 53.8 | 14.9 (10.0) | 41.5 36.1 | 56.6 (19.8) | 104.2 54.3 | 17.3 (17.1) | 65.7 26.3 | 6.5 (1.7) | 10.3 63.5 | 102.6 (25.1) | 8.8 | 12.5 |
| P4 | T1 | 23.9 (10.8) | 45.3 52.7 | 62.6 (30.9) | 146.2 42.8 | 41.3 (22.8) | 97.0 42.6 | 146.4 (87.8) | 443.1 33.0 | 76.3 (37.6) | 162.7 46.9 | 49.7 (29.6) | 115.5 43.0 | 61.3 (22.9) | 5.06 | 7.29 |
|  | T2 | 21.2 (9.0) | 41.0 51.5 | 61.5 (28.3) | 131.5 46.8 | 38.8 (19.2) | 88.8 43.7 | 134.4 (75.7) | 406.8 33.0 | 74.2 (37.6) | 170.1 43.6 | 57.4 (28.5) | 133.3 (43.0) | 68.7 (21.0) | 6.10 | 7.99 |
|  | T3 | 21.2 (9.6) | 44.4 47.7 | 51.3 (23.4) | 116.0 44.2 | 37.9 (17.3) | 75.3 50.4 | 134.5 (62.4) | 334.3 40.2 | 55.8 (31.2) | 139.2 40.1 | 28.5 (13.9) | 56.5 50.5 | 74.9 (19.3) | 4.85 | 5.27 |
| P5 | T1 | 9.4 (3.2) | 17.1 54.9 | 52.5 (18.7) | 97.0 54.1 | 19.1 (6.5) | 32.1 59.5 | 24.4 (9.8) | 50.5 48.2 | 40.1 (24.7) | 107.3 37.4 | 47.6 (17.8) | 95.5 49.8 | 78.8 (11.1) | 3.25 | 4.92 |
|  | T2 | 10.9 (4.6) | 22.5 48.8 | 51.0 (19.1) | 96.1 53.1 | 18.8 (7.4) | 34.9 54.0 | 25.6 (11.0) | 52.4 48.8 | 40.9 (28.9) | 120.3 34.1 | 43.2 (17.9) | 85.6 50.4 | 82.3 (13.7) | 4.09 | 6.71 |
|  | T3 | 9.5 (3.7) | 18.3 51.9 | 54.2 (21.9) | 112.1 48.4 | 15.7 (7.9) | 38.1 (41.1) | 24.7 (9.4) | 50.0 49.4 | 41.0 (28.1) | 106.5 38.5 | 45.3 (17.8) | 86.5 52.3 | 85.4 (12.6) | 4.91 | 7.12 |
| P6 | T1 | 13.6 (5.9) | 28.8 47.3 | 54.9 (33.2) | 137.8 39.9 | 82.9 (30.4) | 154.3 53.8 | 164.2 (82.5) | 441.7 37.2 | 103.7 (42.7) | 197.2 52.6 | 112.1 (32.5) | 187.5 59.8 | 88.0 (8.4) | 2.89 | 5.10 |
|  | T2 | 12.5 (5.8) | 29.2 42.9 | 63.2 (36.3) | 155.9 40.6 | 87.8 (33.7) | 160.1 54.8 | 182.4 (103.5) | 436.9 41.7 | 96.1 (36.9) | 184.8 51.9 | 108.9 (30.1) | 166.8 65.3 | 84.9 (8.6) | 2.99 | 5.33 |
|  | T3 | 12.6 (5.5) | 25.9 48.3 | 59.9 (37.3) | 160.9 37.2 | 83.4 (34.3) | 156.6 53.2 | 164.4 (74.5) | 389.9 42.2 | 84.1 (46.2) | 184.9 45.5 | 78.1 (33.9) | 143.9 54.2 | 85.0 (9.4) | 3.31 | 5.82 |
| P7 | T1 | 26.7 (18.3) | 75.9 35.2 | 57.2 (38.2) | 168.7 33.9 | 26.2 (17.4) | 69.8 37.6 | 74.1 (22.8) | 128.2 57.8 | 33.2 (18.6) | 88.5 37.4 | 13.6 (4.9) | 26.4 51.4 | 52.9 (23.7) | 2.97 | 6.26 |
|  | T2 | 23.4 (16.6) | 70.3 33.2 | 48.7 (30.3) | 125.1 38.9 | 24.4 (16.3) | 61.9 39.5 | 70.4 (25.2) | 138.0 51.0 | 25.6 (16.2) | 75.9 33.8 | 14.2 (6.5) | 35.2 40.3 | 53.2 (23.8) | 2.29 | 5.54 |
|  | T3 | 25.9 (19.3) | 78.8 32.9 | 52.9 (34.3) | 156.7 33.7 | 26.4 (17.1) | 67.5 39.1 | 72.0 (23.7) | 131.3 54.8 | 25.2 (12.5) | 57.4 43.9 | 13.3 (4.9) | 25.2 52.7 | 58.2 (24.6) | 2.10 | 5.45 |
| P8 | T1 | 10.5 (2.6) | 16.6 63.6 | 35.9 (15.9) | 76.3 47.0 | 14.6 (6.5) | 29.1 50.0 | 95.7 (31.9) | 182.9 52.3 | 21.1 (5.0) | 32.2 65.7 | 17.8 (5.9) | 32.0 55.7 | 75.7 (19.2) | 3.62 | 8.37 |
|  | T2 | 7.5 (2.0) | 11.9 62.7 | 30.7 (15.8) | 69.7 44.0 | 13.7 (5.8) | 26.4 51.8 | 92.0 (31.3) | 161.9 56.8 | 18.7 (5.3) | 29.1 64.2 | 15.5 (5.3) | 27.4 56.4 | 65.6 (17.6) | 3.25 | 7.49 |
|  | T3 | 7.1 (2.6) | 14.2 50.2 | 28.1 (13.9) | 67.5 41.6 | 12.6 (5.6) | 25.7 49.1 | 78.1 (26.1) | 139.5 56.0 | 17.9 (3.9) | 25.3 70.7 | 13.9 (4.4) | 25.0 55.8 | 66.9 (17.8) | 3.50 | 8.66 |
| P9 | T1 | 19.3 (9.6) | 40.4 47.7 | 60.9 (30.0) | 151.5 40.3 | 39.2 (14.2) | 72.4 54.1 | 146.4 (52.1) | 317.2 46.1 | 62.4 (18.3) | 105.9 58.8 | 59.1 (37.6) | 172.8 34.2 | 102.5 (11.7) | 2.53 | 4.83 |
|  | T2 | 20.6 (12.5) | 54.7 37.6 | 59.5 (24.6) | 128.9 46.1 | 40.3 (15.1) | 74.9 53.7 | 120.5 (36.7) | 207.7 58.0 | 61.6 (19.2) | 109.1 56.4 | 55.3 (33.9) | 147.7 37.4 | 103.0 (7.7) | 2.49 | 4.83 |
|  | T3 | 22.9 (12.5) | 54.8 41.7 | 56.6 (25.7) | 129.8 43.6 | 40.4 (16.3) | 80.5 50.2 | 115.8 (43.9) | 237.6 48.7 | 63.2 (18.8) | 110.6 (57.2) | 58.1 (39.9) | 187.5 30.9 | 95.5 (7.9) | 2.58 | 5.21 |
| P10 | T1 | 20.3 (6.9) | 38.2 53.1 | 34.0 (15.3) | 67.9 50.0 | 19.8 (8.9) | 46.7 42.3 | 42.2 (14.9) | 79.5 53.0 | 20.9 (7.1) | 36.9 56.8 | 8.4 (3.3) | 15.6 53.5 | 68.5 (6.5) | 2.20 | 5.18 |
|  | T2 | 20.3 (7.9) | 44.4 45.7 | 30.1 (13.8) | 62.8 47.9 | 20.0 (8.8) | 44.6 44.9 | 35.7 (10.3) | 59.5 59.9 | 18.5 (6.7) | 36.3 50.9 | 8.8 (3.1) | 15.8 56.0 | 72.3 (9.4) | 2.90 | 5.83 |
|  | T3 | 20.3 (7.9) | 46.4 43.7 | 30.4 (13.7) | 61.8 49.1 | 19.9 (10.5) | 53.9 36.9 | 27.9 (6.1) | 40.3 69.1 | 18.9 (5.4) | 29.3 64.4 | 7.8 (3.0) | 14.6 53.1 | 60.8 (9.5) | 2.93 | 6.74 |
| P11 | T1 | 15.0 (14.1) | 54.5 27.6 | 44.8 (27.4) | 130.6 34.3 | 34.2 (9.4) | 53.1 64.4 | 88.0 (29.9) | 157.3 55.9 | 43.9 (31.0) | 152.1 28.9 | 30.7 (13.6) | 59.4 51.6 | 139.0 (10.2) | 4.17 | 2.73 |
|  | T2 | 14.3 (13.2) | 51.9 27.4 | 43.6 (25.1) | 115.7 37.7 | 35.8 (11.9) | 64.2 55.8 | 90.9 (32.2) | 164.9 55.1 | 42.4 (27.7) | 124.7 33.9 | 31.9 (14.3) | 69.4 45.9 | 123.1 (9.2) | 3.95 | 3.22 |
|  | T3 | 13.6 (12.7) | 49.8 27.3 | 40.9 (24.2) | 112.7 36.3 | 31.2 (13.0) | 56.9 55.0 | 80.3 (28.8) | 150.9 53.2 | 34.4 (21.9) | 98.4 34.9 | 30.0 (11.0) | 49.6 60.5 | 139.4 (10.0) | 4.35 | 2.89 |
| P12 | T1 | 16.3 (9.5) | 44.5 36.6 | 42.6 (19.4) | 87.9 48.5 | 27.6 (11.4) | 56.5 48.9 | 85.7 (30.6) | 158.8 53.9 | 35.1 (8.0) | 55.7 63.1 | 39.5 (9.7) | 59.3 66.6 | 70.1 (10.6) | 2.36 | 3.58 |
|  | T2 | 15.7 (8.2) | 34.4 45.6 | 35.1 (14.5) | 71.3 49.3 | 25.1 (8.5) | 41.4 60.7 | 74.9 (29.1) | 141.8 52.8 | 30.6 (7.7) | 52.9 57.8 | 42.1 (11.4) | 69.1 60.9 | 79.7 (9.8) | 2.17 | 2.95 |
|  | T3 | 16.1 (8.7) | 38.8 41.5 | 35.9 (15.4) | 76.9 46.6 | 26.4 (8.9) | 49.5 53.3 | 84.5 (34.9) | 186.7 45.3 | 27.6 (6.8) | 42.9 64.3 | 35.9 (9.5) | 56.7 63.3 | 76.7 (9.3) | 2.02 | 3.22 |
| P13 | T1 | 16.0 (9.2) | 39.1 40.9 | 39.5 (13.7) | 70.8 55.8 | 49.6 (27.3) | 107.8 46.0 | 104.4 (30.2) | 171.8 60.7 | 119.0 (49.2) | 213.2 55.8 | 27.6 (21.5) | 78.3 35.4 | 78.6 (17.3) | 4.23 | 7.92 |
|  | T2 | 14.7 (9.1) | 38.8 38.1 | 37.3 (11.5) | 63.5 58.6 | 53.4 (25.8) | 111.1 48.1 | 87.4 (24.8) | 137.5 63.5 | 114.4 (52.9) | 221.7 51.6 | 20.8 (18.4) | 89.1 23.4 | 100.9 (14.1) | 3.79 | 6.52 |
|  | T3 | 11.9 (7.3) | 30.0 39.8 | 31.0 (10.8) | 54.2 57.2 | 43.9 (21.0) | 84.7 51.9 | 93.3 (32.4) | 171.1 54.5 | 89.3 (39.9) | 168.9 52.8 | 36.9 (22.4) | 84.3 43.8 | 94.9 (12.6) | 3.74 | 5.91 |
| P14 | T1 | 26.7 (18.7) | 88.2 30.4 | 55.2 (23.9) | 126.5 43.6 | 27.9 (15.8) | 64.4 43.3 | 84.7 (40.3) | 176.8 47.8 | 47.8 (18.6) | 88.7 53.9 | 32.3 (19.7) | 71.6 45.1 | 93.7 (12.6) | 2.73 | 5.56 |
|  | T2 | 20.2 (13.5) | 68.7 29.4 | 46.4 (20.7) | 94.4 49.2 | 25.6 (15.6) | 73.1 35.1 | 86.2 (46.7) | 219.3 39.3 | 43.3 (15.6) | 83.6 51.8 | 25.5 (13.9) | 58.9 43.2 | 90.1 (11.7) | 2.82 | 6.07 |
|  | T3 | 18.3 (11.5) | 55.1 33.2 | 45.9 (19.9) | 99.6 46.2 | 25.2 (14.6) | 67.2 37.6 | 68.7 (34.5) | 162.9 42.2 | 37.8 (18.1) | 89.4 42.3 | 13.5 (12.8) | 54.7 24.7 | 90.9 (10.9) | 3.01 | 6.45 |
| total | T1 | 18.3 (9.6) | 43.1 45.8 | 46.5 (22.8) | 107.4 44.8 | 30.9 (14.7) | 67.5 45.5 | 95.2 (38.5) | 204.4 51.1 | 53.9 (24.1) | 111.6 49.2 | 38.2 (17.7) | 80.6 49.6 | 84.0 (14.0) | 3.73 (1.49) | 6.15 (2.25) |
|  | T2 | 16.8 (10.1) | 45.1 40.9 | 43.2 (21.1) | 96.6 45.3 | 31.0 (15.3) | 69.2 45.4 | 88.5 (38.5) | 189.1 49.9 | 50.9 (24.9) | 111.5 46.3 | 37.6 (17.4) | 80.7 47.5 | 83.9 (13.9) | 3.59 (1.36) | 6.19 (2.14) |
|  | T3 | 16.4 (9.1) | 40.9 41.9 | 41.9 (20.3) | 96.3 44.9 | 29.0 (14.1) | 63.4 44.2 | 87.5 (33.9) | 181.4 50.8 | 44.6 (22.0) | 96.9 48.2 | 31.5 (14.9) | 67.4 50.0 | 83.8 (14.0) | 3.99 (1.89) | 6.54 (2.55) |
